# Maternal Methadone Destabilizes Neonatal Breathing and Desensitizes Neonates to Opioid-Induced Respiratory Frequency Depression

**DOI:** 10.3389/fphys.2021.604593

**Published:** 2021-02-25

**Authors:** Austin D. Hocker, Nina R. Morrison, Matthew L. Selby, Adrianne G. Huxtable

**Affiliations:** Department of Human Physiology, University of Oregon, Eugene, OR, United States

**Keywords:** opioids, maternal insults, neonatal, breathing, chemosensitivity, opioid-induced respiratory frequency depression, quantal slowing

## Abstract

Pregnant women and developing infants are understudied populations in the opioid crisis, despite the rise in opioid use during pregnancy. Maternal opioid use results in diverse negative outcomes for the fetus/newborn, including death; however, the effects of perinatal (maternal and neonatal) opioids on developing respiratory circuitry are not well understood. Given the profound depressive effects of opioids on central respiratory networks controlling breathing, we tested the hypothesis that perinatal opioid exposure impairs respiratory neural circuitry, creating breathing instability. Our data demonstrate maternal opioids increase apneas and destabilize neonatal breathing. Maternal opioids also blunted opioid-induced respiratory frequency depression acutely in neonates; a unique finding since adult respiratory circuity does not desensitize to opioids. This desensitization normalized rapidly between postnatal days 1 and 2 (P1 and P2), the same age quantal slowing emerged in respiratory rhythm. These data suggest significant reorganization of respiratory rhythm generating circuits at P1–2, the same time as the preBötzinger Complex (key site of respiratory rhythm generation) becomes the dominant respiratory rhythm generator. Thus, these studies provide critical insight relevant to the normal developmental trajectory of respiratory circuits and suggest changes to mutual coupling between respiratory oscillators, while also highlighting how maternal opioids alter these developing circuits. In conclusion, the results presented demonstrate neurorespiratory disruption by maternal opioids and blunted opioid-induced respiratory frequency depression with neonatal opioids, which will be important for understanding and treating the increasing population of neonates exposed to gestational opioids.

## Key points summary

• Pregnant women and developing infants are understudied populations in the opioid crisis, despite the rise in opioid use during pregnancy.
• Opioids have profound depressive effects on the control of breathing, yet the effects of perinatal (maternal and neonatal) opioids on the development of respiratory control networks are unknown.
• We tested the hypothesis that late gestation perinatal opioids impair respiratory neural circuitry, creating breathing instability
• Our data demonstrate maternal opioids increase apneas and destabilize neonatal breathing.
• Maternal opioids blunt opioid-induced respiratory frequency depression acutely in neonates; a unique finding since adult respiratory circuitry generally does not sensitize to opioids.
• Blunted opioid-induced respiratory frequency depression occurs at a key time point for reorganization of central respiratory circuits.
• This study highlights the effects of maternal opioids on developing respiratory circuits and provides critical insight relevant to the normal developmental trajectory of respiratory circuits.

## Introduction

The misuse of opioids is a national and public health crisis with greater than 118 Americans dying from opioid overdose daily in 2016, 27% more deaths than the previous year (Haight et al., 2018). This crisis carries a significant economic burden, estimated to be $78.5 billion/year (Florence et al., 2016). Significant efforts are underway to curb the misuse of opioids in adults, yet one understudied population in these efforts is pregnant women. In fact, opioid use during pregnancy is on the rise (Epstein et al., 2013; Krans et al., 2015; Stover and Davis, 2015) and a significant need exists to improve treatments for neonates after maternal opioids (Kraft et al., 2016). Maternal opioids lead to neonatal abstinence syndrome (NAS), described as dysfunction in the central and autonomic nervous system, and gastrointestinal system in infants (McQueen and Murphy-Oikonen, 2016). Further, NAS induces diverse negative outcomes in infants, including; irritability, sleep disturbances, tachypnea, respiratory disturbances, and death (McQueen and Murphy-Oikonen, 2016). However, the respiratory disturbances are not well characterized, but increased apneas have been described (Ward et al., 1986; McQueen and Murphy-Oikonen, 2016). Despite this increasing population of NAS infants, little is known about the effects of maternal opioids on developing neonatal respiratory control circuitry.

At birth, neonates must have a functional respiratory system capable of robust, rhythmic breathing. Therefore, rhythmic neural activity, initiating at the preBötzinger complex (preBötC, the kernel for respiratory rhythmogenesis) (reviewed in Del Negro et al., 2018) is required for development of neural circuitry, respiratory muscles, and the lungs (Pagliardini et al., 2003; Greer, 2012). In rats, development of rhythmogenic respiratory neural networks begins around embryonic day 17 (E17) and continues until birth (Pagliardini et al., 2003). The preBötC is exceptionally sensitive to opioids and is responsible for many of the respiratory depressant effects of opioids (Greer et al., 1995; Ren et al., 2006; Montandon et al., 2011, 2016; Stucke et al., 2015). Since maternal opioids cross the placenta and delay neurodevelopment (Kopecky et al., 1999; Hauser and Knapp, 2018), they have the potential to suppress rhythmic respiratory activity from the preBötC after preBötC neurogenesis is complete. Thus, respiratory control networks need to compensate to maintain adequate respiratory function at birth (Gourévitch et al., 2017). Interestingly, the parafacial respiratory group (pFRG) also has rhythmogenic properties during embryonic and early neonatal life (Onimaru and Homma, 2003; Thoby-Brisson et al., 2009) and may be able to mitigate respiratory instability during early life (Feldman and Del Negro, 2006). However, controlled studies on neonatal breathing after maternal opioids are limited. One study demonstrated medullary respiratory network reorganization after maternal opioid exposure in neonatal rats (Gourévitch et al., 2017). Another study in guinea pigs shows enhanced hypercapnic ventilatory responses (HCVR) after prenatal opioids (Nettleton et al., 2008), suggesting changes in chemosensitivity after maternal opioids. To our knowledge, this is the first study investigating neonatal breathing after perinatal opioids in rats. For this study, we tested the effects of maternal methadone (MM) to model aspects of maternal opioid use since methadone is a standard treatment for maternal opioid dependence (Bhavsar et al., 2018). Since we were primarily interested in the effects of opioids on maturation of respiratory networks (rather than effects on neurogenesis, neuronal migration, differentiation, and oligodendrogenesis), we investigated the effects of opioids at the onset of respiratory rhythmogenesis. Additionally, NAS infants are treated acutely with opioids, such as methadone, to alleviate NAS symptoms (Kraft et al., 2016; Pryor et al., 2017; Davis et al., 2018). But since opioids dangerously depress breathing in neonates and may cause lasting neural deficits (Attarian et al., 2014), we hypothesized that repetitive, daily acute methadone would further destabilize breathing after MM.

Understanding the acute effects of perinatal opioid use is critical to developing more effective treatments for neonates exposed to perinatal opioids. We hypothesize that perinatal methadone exposure during development of the respiratory control system will destabilize neonatal breathing, blunt hypoxic ventilatory responses (HVR), and enhance HCVR. Understanding how perinatal opioid use alters the respiratory response to acute opioids will contribute to our understanding of how to alleviate the symptoms of NAS without severe respiratory dysfunction.

## Materials and Methods

All experiments conformed to the policies of the National Institutes of Health *Guide for the Care and Use of Laboratory Animals* and were approved by the Institutional Animal Care and Use Committee at the University of Oregon. Timed pregnant Sprague Dawley rats (Envigo Colony 217) were ordered to arrive at gestational day 17 (or embryonic day 17, E17, for the fetuses) and were housed under standard conditions with a 12:12 h light/dark cycle with food and water *ad libitum*.

### Maternal Opioid Exposure

**FIGURE 1:**
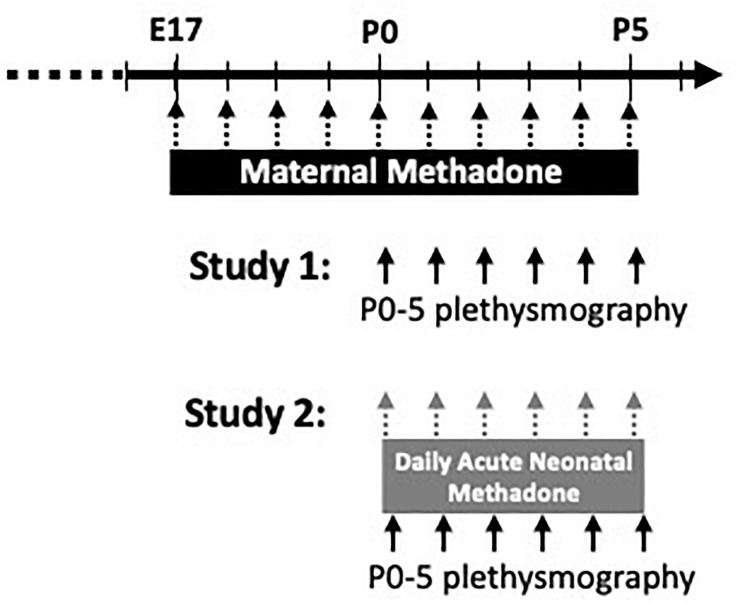
Schematic of experimental paradigm. Pregnant dams were treated from E17-P5 with daily maternal methadone injections (5 mg/kg, s.c.). Control dams were injected with vehicle (saline) or untreated. In study 1, baseline breathing, hypercapnic, and hypoxic ventilatory responses in neonates (P0–5) from each maternal treatment group were assessed by whole-body plethysmography. In study 2, neonates from each maternal treatment group were given daily acute, neonatal methadone (1 mg/kg, i.p.) from P0 to P5. Baseline breathing, ventilatory responses to neonatal methadone, and hypoxic and hypercapnic ventilatory responses were assessed at P0–5 by whole-body plethysmography.

Since respiratory rhythm generation and fetal breathing movements begin on day E17 in fetal rats (Pagliardini et al., 2003; Greer, 2012), MM exposure began on E17 to mitigate other developmental effects of opioids and focus the effects of opioids on developing respiratory circuitry. Each morning from E17 onward, dams were injected with methadone (5 mg/kg in sterile saline, Sigma-Aldrich, subcutaneous) and monitored for at least 1 h. After parturition, daily MM injections continued until postnatal day 5 (P5) (Figure 1). Control dams were injected daily with vehicle (sterile saline and subcutaneous) or were un-treated to control for the potential stress of daily injections. Litters were culled to 12 or fewer pups per dam to ensure each dam fostered the same number of pups. Breathing in neonates (P0–5) born to MM, maternal saline (MS), and maternal no-treatment (MN) were studied using a custom-built plethysmograph.

In the first study, neonatal baseline ventilation, hypoxic, and HCVR were assessed from P0 to P5 (Figure 1). Since no differences were found between sexes (*p* > 0.05), male and female rats were combined for MM (MM: P0 = 8 female, 8 male, P1 = 8 female, 4 male, P2 = 9 female, 4 male, P3 = 7 female, 4 male, P4 = 7 female, 2 male, P5 = 7 female, 2 male), maternal saline (MS: P0 = 8 female, 5 male, P1 = 8 female, 7 male, P2 = 8 female, 7 male, P3 = 5 female, 6 male, P4 = 5 female, 5 male, P5 = 5 female, 6 male), and maternal no treatment (MN: P0 = 10 female, 12 male, P1 = 7 female, 11 male, P2 = 4 female, 6 male, P3 = 7 female, 9 male, P4 = 6 female, 8 male, P5 = 7 female, 7 male).

In a separate group of neonates in the second study, the combined effects of maternal treatment with daily acute, neonatal methadone (NM, 1 mg/kg, i.p.) in neonates from P0 to P5 were assessed (Figure 1). Injections began at P0 and were repeated daily until P5. Thus, each neonate received a total of 6 NM injections by P5. For each neonate, baseline breathing was measured for 10 min before an acute methadone injection. The ventilatory response to NM was monitored for 1 h, followed by hypercapnic and HVR. Since no differences were found between sexes, male and female rats exposed to NM were combined from each maternal treatment (MM: P0 = 7 female, 7 male, P1 = 6 female, 8 male, P2 = 8 female, 5 male, P3 = 8 female, 7 male, P4 = 8 female, 7 male, P5 = 7 female, 7 male; MS: P0 = 4 female, 5 male, P1 = 3 female, 6 male, P2 = 2 female, 7 male, P3 = 2 female, 7 male, P4 = 3 female, 5 male, P5 = 5 female, 4 male; MN: P0 = 2 female, 2 male, P1 = 2 female, 4 male, P2 = 2 female, 4 male, P3 = 2 female, 4 male, P4 = 2 female, 4 male, P5 = 2 female, 2 male).

### Neonatal Whole-Body Plethysmography

Ventilation was measured in freely behaving neonates from P0 to P5 using custom built whole-body plethysmography designed and constructed by Dr. J. J. Greer and colleagues (Ren et al., 2012, 2013, 2019). Neonatal rats were individually placed in an acrylic chamber (75 ml volume). Three mass flow controllers (Alicat Scientific) controlled the concentration of oxygen, nitrogen, and carbon dioxide at a total flow rate of 100 ml/min. Body surface temperature was maintained throughout experiments at thermoneutral (Eden and Hanson, 1987; Mortola and Dotta, 1992) by adjusting chamber temperature between 32–34°C with a heating pad (Kent Scientific). A differential pressure transducer (ADInstruments^®^), attached to the chamber via a dedicated port, recorded pressure changes due to ventilation relative to pressure in an identical reference chamber. Data were recorded using LabChart software (PowerLab System, ADInstruments^®^/LabChart, v.8) using a low-pass filter (30 Hz).

### Hypoxic and Hypercapnic Ventilatory Responses

Hypercapnic ventilatory responses and HVR were measured daily (P0–5). After a baseline recording period (10–40 min, 21% O_2_, balance N_2_), inflow gas switched to 5% CO_2_ (10 min) to assess HCVR. The HCVR response was assessed during the last 2 min of the hypercapnic stimulus and made relative to baseline *V*_E_ taken during the preceding 5 min before hypercapnic exposure. After hypercapnia, chambers were returned to normocapnia (21% O_2_, balance N_2_, 20 min) for neonates to recover (20 min). To evaluate HVR, inflow gas was switched to 10% oxygen (10 min). Peak, phase I, HVRs were calculated as the maximum rolling average of 50 breaths during the hypoxic stimulus and made relative to baseline *V*_E_ in the 5 min immediately preceding hypoxia. Phase II, hypoxic ventilatory declines were assessed as the average *V*_E_ in the last 2 min of the hypoxic exposure and made relative to baseline *V*_E_ in the 5 min immediately preceding hypoxia.

### Methadone-Induced Respiratory Frequency Depression and Quantal Slowing

Respiratory responses to acute neonatal methadone were measured daily in neonates (P0–5) after MM, MS, and MN. Neonates were placed in the recording chamber for baseline recordings (10 min). Neonates were removed from the chamber, injected with methadone (1 mg/kg, i.p.) and immediately returned the chamber for continuous recording (1 h). HCVRs and HVRs were measured 1 h after acute methadone injections, as described above.

Quantal slowing is the reduction of breathing frequency by integer multiples of the basal breathing period in response to acute opioids (Mellen et al., 2003). To assess quantal slowing patterns after acute methadone, Poincaré plots of normalized breathing periods were used to identify the presence of breaths clustered at integers of breathing period. Poincaré plots graph the period between two breaths (*T*_n_) versus the subsequent period (*T*_n+1_). These were generated in R using a custom script and overlay from periods obtained from LabChart (v8, AD Instruments^®^).

### Data Analysis

For all experiments, inspiratory frequency and *V*_T_ were analyzed using peak analysis in LabChart (v8, AD Instruments^®^). Two respiratory variables, tidal volume (*V*_T_) and frequency (breaths/minute) are estimated from the amplitude and the frequency of the pressure recordings. Minute ventilation (*V*_E_) is calculated as the product of frequency and *V*_T_. *V*_T_ and *V*_E_ were normalized to body weight (g). The custom-built plethysmograph is primarily effective for studying respiratory frequency and is not designed for absolute quantification of *V*_T_. Therefore, *V*_T_ and *V*_E_ are reported as changes relative to baseline when assessing HVR, HCVR, and changes in breathing after neonatal methadone.

Apneas were identified as respiratory pauses lasting longer than 2.5 breaths compared to the baseline frequency. Post-sigh apneas were defined as an augmented breath with at least twice the mean *V*_T_, followed by an apnea (Marcouiller et al., 2014). To assess respiratory rhythm variability, short-term (SD1) and long-term (SD2) variability were calculated from breath-to-breath intervals during baseline in R using a custom script.

Statistical comparisons and visualization for all data were performed using Graphpad Prism (version 8.4.0), in addition to R and R studio (version 1.1.463). Differences between means were identified using mixed-model two-way ANOVA with Bonferroni correction for multiple comparisons (α = 0.05). Values are reported as means ± SD.

## Results

Sex had no effect on any respiratory variables (*p* > 0.05); thus, male and female rats were combined for all analyses. Neonatal body weights at each age from P0 to P5 were largely similar, regardless of maternal treatment (Table 1). As expected, neonatal body weights significantly increased from P0 to P5 (*p* < 0.0001), with the exceptions of no significant changes from P0 to P1 or P4 to P5 after MM, and P2 to P3 after MN.

**TABLE 1:** Maternal methadone does not alter neonatal weight.

	**P0**	**P1**	**P2**	**P3**	**P4**	**P5**
MM	5.3 ± 0.6	6.2 ± 0.5	7.0 ± 0.6 *	8.7 ± 0.6^@^	10.1 ± 0.6	11.2 ± 1.0
MS	5.2 ± 0.4	5.8 ± 0.4	6.5 ± 0.6 ^#^	7.7 ± 0.7	9.4 ± 0.5	10.8 ± 1.0
MN	5.5 ± 0.5	6.0 ± 0.7	8.0 ± 0.5	8.1 ± 0.9	9.1 ± 1.4	10.5 ± 1.4

Mean ± SD; mixed-model ANOVA, Bonferroni *post hoc*, *MM *p* < 0.001 from MN, ^#^MS *p* < 0.0001 from MN, ^@^MM *p* < 0.01 from MS.

### Maternal Methadone Destabilizes Neonatal Respiratory Rhythm at P0.

**FIGURE 2:**
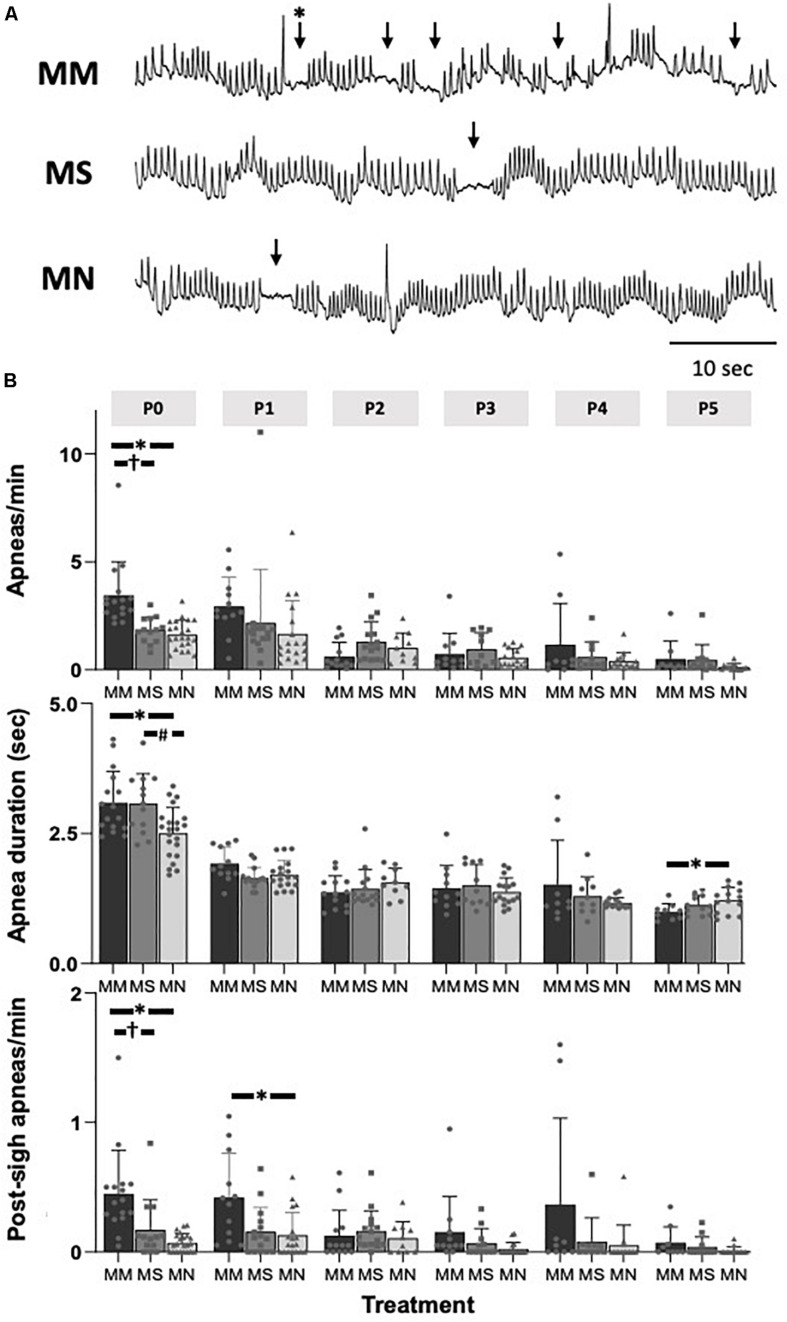
Maternal methadone increases apneas at P0. Representative baseline plethysmography traces **(A)** show frequent apneas (↓) and post-sigh apneas (^∗^↓) after maternal methadone (MM, dark bars). Group data demonstrate MM increases the frequency of apneas at P0 compared to neonates after maternal saline (MS, gray bars) and maternal no-treatment (MN, light gray bars) **(B, top)**. MM and MS increased the duration of apneas compared to MN **(B**, **middle)**. MM also increased the frequency of post-sigh apneas compared to MS and MN neonates at P0 and P1 **(B, bottom)**. By P1, the frequency and duration of apneas were not different between treatment groups. Mixed-model ANOVA, Bonferroni *post hoc*, ^∗^MM *p* < 0.05 from MN, ^†^MM *p* < 0.05 from MS, ^#^MN *p* < 0.05 from MS.

At P0, apneas were significantly more frequent after MM (*p* < 0.01 compared to MS and MN, Figures 2A,B), indicating less stable breathing after MM. Apnea duration was greater in P0 neonates after MM and MS compared to MN (*p* = 0.012 and *p* = 0.021, respectively, Figure 2B), and slightly, but significantly less in P5 neonates compared to MN (*p* = 0.045). Additionally, post-sigh apneas were more frequent in P0 neonates after MM compared to MS (*p* = 0.04) and MN (*p* = 0.001) and in P1 neonates after MM compared to MN (*p* = 0.047). At P0, MM treated neonates had more sighs (4.1 ± 1.7 min^–1^) compared to MN (1.1 ± 0.5 min^–1^, *p* < 0.0001) and MS (1.6 ± 1.0 min^–1^, *p* = 0.0001). Additionally, neonates after MM had more sighs at P1 (4.3 ± 2.5, *p* = 0.02) and P3 (3.9 ± 2.4, *p* = 0.04) compared to neonates after MN (P1: 1.7 ± 1.2; P3: 1.6 ± 1.1). At P5, neonates after MS (5.4 ± 3.4) had more sighs than neonates after MN (1.3 ± 0.8, *p* = 0.07). The fraction of sighs followed by an apnea was not significantly different between groups at any age (*p* > 0.05).

Regardless of maternal treatment, overall baseline breathing was similar between groups, except at P0 (Table 2). Additionally, frequency at P1 after MM was increased compared to MS neonates (*p* = 0.016) (Table 2). At P5, breathing frequency after MM was increased compared to MN neonates (*p* = 0.01), but not MS (Table 2). MM increased *V*_T_ at P0 compared to MS (*p* < 0.001) and MN (*p* = 0.001), but *V*_T_ normalized by P1 (Table 2). *V*_T_ in MS neonates at P4 was decreased compared to both neonates after MM (*p* = 0.017) and after MN (*p* = 0.02). *V*_E_ also increased at P0 after MM compared to MS (*p* < 0.0001) and MN (*p* = 0.0013, Table 2).

**TABLE 2:** Maternal methadone disrupts baseline breathing at P0.

		**P0**	**P1**	**P2**	**P3**	**P4**	**P5**
Frequency (breaths/min)	MM	120.327.3^†^	155.614.9^†^	168.830.1	165.418.7	173.229	202.224.8*
	MS	96.517.1	138.014.6	163.619.4	155.617	173.821.9	183.516.2
	MN	104.718.7	141.614.2	150.911.4	157.614.9	178.612.6	166.125.7
*V*_T_ (ml/100 g)	MM	0.810.15^*†^	0.820.19	0.680.16	0.680.17	0.790.16^†^	0.840.21
	MS	0.510.10	0.780.15	0.720.16	0.670.05	0.580.09	0.650.13
	MN	0.580.15	0.730.10	0.600.08	0.660.15	0.770.22^#^	0.740.23
*V*_E_ (ml/min/100 g)	MM	98.130.4^*†^	127.829.2	117.545.0	113.435.5	136.838.8	173.867.4
	MS	49.815.3	108.221.2	119.132.3	103.916.6	101.425.0	119.831.3
	MN	61.224.2	103.716.3	92.115.1^#^	104.728.9	138.742.8^#^	12654.3
Frequency CV	MM	0.920.23*	0.640.23^*†^	0.290.11	0.350.19	0.430.42	0.290.07
	MS	0.730.20	0.420.07	0.400.17	0.350.14	0.310.1	0.290.10
	MN	0.550.13^#^	0.420.16	0.370.11	0.290.1	0.270.13	0.240.07
SD1	MM	0.480.15^∗^	0.210.05	0.130.03	0.140.05	0.180.14	0.100.01*
	MS	0.500.15	0.180.03	0.160.08	0.150.03	0.130.03	0.120.02
	MN	0.340.12^#^	0.180.04	0.150.02	0.130.02	0.120.02	0.130.02
SD2	MM	0.570.19*	0.220.08	0.110.06	0.140.10	0.190.23	0.110.03
	MS	0.540.21	0.160.04	0.140.09	0.130.06	0.120.05	0.110.05
	MN	0.340.14^#^	0.160.06	0.140.05	0.110.04	0.110.05	0.110.04

Mixed-model ANOVA, Bonferroni *post hoc*, *MM *p* < 0.05 from MN, ^†^MM *p* < 0.05 from MS, ^#^MN *p* < 0.05 from MS.

Corresponding to the relative instability at P0 after MM, frequency CV was increased at P0 after MM compared to MN (*p* < 0.0001, Table 2). Additionally, SD1 and SD2 were elevated in MM and MS at P0 compared to MN (*p* < 0.001, Table 2), further supporting destabilization of breathing after MM and suggesting MS may also increase variability in early neonatal breathing pattern. Alterations in breathing after MS may reflect the effects of maternal stress (due to repeated injections) on neonatal breathing. Breathing variability after MM normalized with age and even showed reduced SD1 at P5 (*p* = 0.033) compared to MN.

### Maternal Methadone Mitigates the Hypoxic Ventilatory Decline at P0, but Does Not Alter Peak HVR or HCVR

**FIGURE 3:**
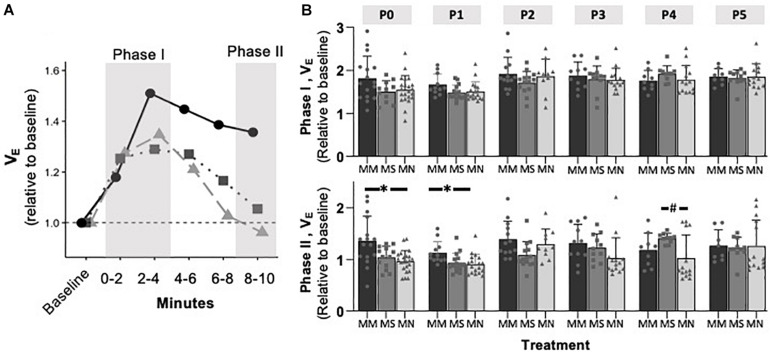
Maternal methadone blunts phase II, hypoxic ventilatory depression at P0 and P1. Average *V*_E_ at P1 for neonates after maternal methadone (MM, dark circles, and bars), maternal saline (MS, gray boxes, and bars), and maternal no-treatment (MN, light gray triangles, and bars) **(A)**. Phase I, peak hypoxic ventilatory responses **(B, top)** were unaffected by maternal treatment group. However, the phase II hypoxic ventilatory decline **(B, bottom)** was blunted at P0 and P1 after MM compared to MN, suggesting impaired central hypoxic responses. Mixed-model ANOVA, Bonferroni *post hoc*, ^∗^MM *p* < 0.05 from MN, ^#^MN *p* < 0.05 from MS.

**FIGURE 4:**
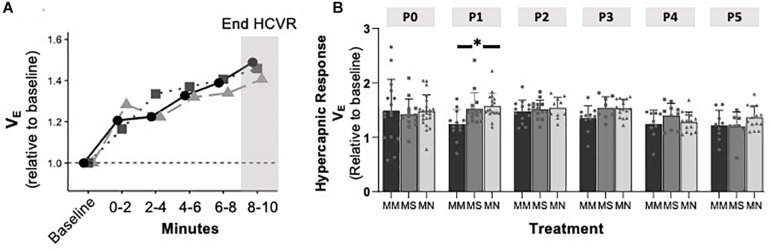
Maternal methadone does not alter hypercapnic ventilatory responses (HCVRs). Average *V*_E_ for P0 maternal methadone (MM, dark circles, and bars), maternal saline (MS, gray squares, and bars), and maternal no-treatment (MN, light gray triangles, and bars) neonates highlight normal hypercapnic responses after MM **(A)**. HCVRs in MM neonates were reduced compared to MN neonates at P1, but no other differences in HCVRs after MM, MN, and MS neonates were evident at other ages **(B)**. Mixed-model ANOVA, Bonferroni *post hoc*, ^∗^ MM *p* < 0.05 from MN.

To assess if MM altered chemosensitivity, the relative ventilatory responses to 10% O_2_ or 5% CO_2_ were assessed. Phase I, peak ventilatory responses were not different between maternal treatment groups (*p* = 0.095), but increased with age (*p* = 0.0001) (Figure 3). However, the ANOVA main effect revealed significant effects of both maternal treatment (*p* = 0.013) and neonatal age (*p* = 0.0005) in the phase II hypoxic ventilatory decline. Pairwise differences showed the phase II hypoxic ventilatory decline was blunted at P0 (*p* = 0.015) and P1 (*p* = 0.024) in MM compared to MN, but not compared to MS (P0 *p* = 0.07, P1 *p* = 0.057) (Figures 3A,B). No pairwise differences in phase I or phase II HVRs were evident after P1, with the exception of reduced phase II in MN neonates at P4 compared to MS neonates (*p* = 0.023). The ventilatory response to 5% CO_2_ was significantly influenced by both maternal treatment (*p* = 0.017) and neonatal age (*p* = 0.004), but there were few pairwise differences. At P1, neonates after MM had a reduced hypercapnic response compared to neonates after MN (*p* = 0.007), but not MS (*p* = 0.052) (Figure 4B). No other pairwise differences were evident at other age groups (Figures 4A,B).

### Daily Neonatal Methadone Blunts Weight Gain

In a separate group of rats from each maternal treatment group, neonates received daily injections of methadone (1 mg/kg, i.p.) starting at P0. Maternal treatment (*p* < 0.0001) and age (*p* < 0.0001) significantly impacted weight. Neonates after MM and neonatal methadone were consistently smaller than neonates after MN and neonatal methadone at all ages (*p* < 0.05, Table 3). After receiving two (P1), four (P3), or five (P4) treatments with daily neonatal methadone, weight was blunted in P1 MM (*p* = 0.01), P3 MM (*p* = 0.0005) and P4 MM (*p* = 0.0048) neonates compared to MS (Table 3). Differences between MS and MN neonates were only evident at P0 (*p* = 0.0002) and P1 (*p* = 0.004).

**TABLE 3:** Daily neonatal methadone reduces weight gain in neonates after maternal methadone.

	**P0**	**P1**	**P2**	**P3**	**P4**	**P5**
MM	5.2 ± 0.7*	5.6 ± 0.6*^†^	6.7 ± 0.7*	7.3 ± 0.9*^†^	8.6 ± 1.0*^†^	10.0 ± 1.2*
MS	5.6 ± 0.4	6.3 ± 0.4	7.2 ± 0.6	8.6 ± 0.6	10.3 ± 0.9	11.4 ± 1.5
MN	6.6 ± 0.2^#^	7.2 ± 0.4^#^	7.6 ± 0.6	8.6 ± 0.9	9.9 ± 0.7	11.8 ± 0.8

Mean ± SD; mixed-model ANOVA, Bonferroni *post hoc*, *MM *p* < 0.05 from MN, ^†^MM *p* < 0.05 from MS, ^#^MN *p* < 0.05 from MS.

### Daily Neonatal Methadone Does Not Alter Neonatal Breathing After Maternal Methadone Exposure

Daily neonatal methadone had little effect on baseline breathing in neonates regardless of maternal treatment (Table 4). Further, analysis of the main effects from daily neonatal methadone demonstrate no significant differences in the frequency of apneas (*p* = 0.33), average apnea duration (*p* = 0.13), or frequency of post-sigh apneas (*p* = 0.40) between maternal treatment groups at baseline, which, contrary to our hypothesis, demonstrates daily neonatal methadone did not further destabilize neonatal breathing. Baseline frequency (*p* = 0.43), *V*_T_ (*p* = 0.11), *V*_E_ (*p* = 0.12), or SD1 (*p* = 0.48) were not different between maternal treatment groups after daily neonatal methadone, demonstrating baseline ventilation is not impacted by daily neonatal methadone. There was a main effect of maternal treatment on SD2 (*p* = 0.0024), whereby SD2 in MM neonates was significantly less at P1 compared to MS (*p* = 0.001) and at P2 compared to MN (*p* = 0.04).

**TABLE 4:** Daily neonatal methadone does not change baseline breathing.

		**P1**	**P2**	**P3**	**P4**	**P5**
Apneas/min	MM	3.72.5	0.80.6	1.62.8	1.00.9	0.60.8
	MS	3.22.3	5.810.2	1.20.8	0.60.6	1.41.8
	MN	5.14.3	2.03.0	0.90.7	1.01.3	0.60.3
Apnea Duration (sec)	MM	2.00.4	1.30.2	1.30.4	1.10.2	1.00.4
	MS	2.31.0	1.80.6	1.40.3	1.20.3	1.20.6
	MN	1.70.5	1.50.3	1.40.5	1.30.3	1.50.6
Post-sigh Apneas/min	MM	0.50.6	0.10.1	0.10.2	0.10.2	0.10.2
	MS	0.30.3	0.92.0	0.20.1	0.00.1	0.30.5
	MN	0.60.7	0.30.6	0.00.1	0.10.1	0.00.1
Frequency (Breaths/min)	MM	149.523.4	176.919.2^†^	172.220.8	192.018.8	204.040.0
	MS	141.422.9	147.824.1	166.526.4	187.427.5	210.830.3
	MN	156.719.0	159.713.0	168.337.1	171.138.8	203.65.7
*V*_T_ (ml/100 g)	MM	0.820.13^†^	0.810.17	0.710.15	0.860.20	0.790.19
	MS	0.680.10	0.760.19	0.710.09	0.690.11	0.800.14
	MN	0.840.07^#^	0.680.10	0.750.15	0.660.21	0.600.10^#^
*V*_E_ (ml/min/100 g)	MM	122.528.7	144.542.7	123.231.9	164.741.5	15837.5
	MS	97.024.9	114.240.2	118.727.1	13132.8	170.243.1
	MN	131.718.7^#^	108.721.5	128.647.2	116.456.8	122.320.4
SD1	MM	0.250.08	0.120.02	0.130.03	0.120.02	0.120.05
	MS	0.230.10	0.200.11	0.140.03	0.110.02	0.110.04
	MN	0.210.09	0.150.04	0.140.04	0.170.07	0.110.01
SD2	MM	0.330.11^†^	0.510.29*	0.70.32	0.650.35	0.570.25
	MS	0.830.27	0.780.44	0.650.37	0.440.18	0.860.69
	MN	0.860.56	1.210.48	1.130.94	1.581.28	0.720.34

To measure the cumulative effects of daily neonatal methadone, baseline breathing was assessed prior to acute neonatal methadone on each day (e.g., P1 neonates received acute methadone on P0 and P5 neonates received daily acute methadone on P0–4). Mixed-model ANOVA, Bonferroni *post hoc*, *MM *p* < 0.05 from MN, ^†^MM *p* < 0.05 from MS, ^#^MN *p* < 0.05 from MS.

### Maternal Methadone Desensitizes P0–1 Neonates From Acute Neonatal Methadone-Induced Frequency Depression

**FIGURE 5:**
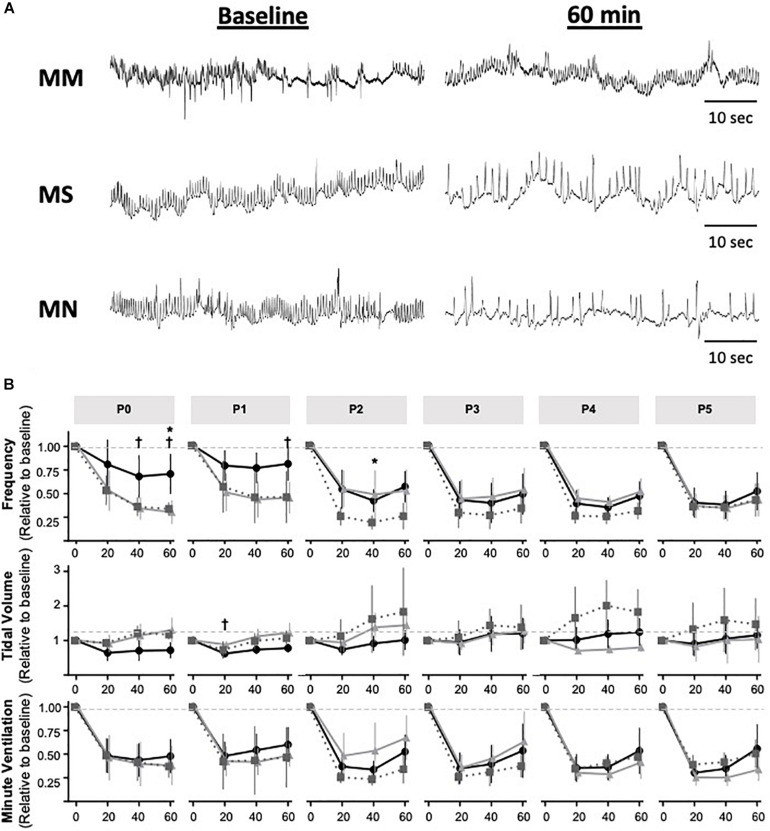
Maternal methadone blunts opioid-induced respiratory frequency depression at P0 and P1. Representative plethysmography traces **(A)** before (baseline) and 60 min after acute methadone at P0. Group data for average breathing frequency, *V*_T_ and *V*_E_ after maternal methadone (MM dark circles), maternal no-treatment (MN gray squares), and maternal saline (MS light grey triangles) combined with daily acute neonatal methadone (1 mg/kg, i.p.). Relative breathing frequency and *V*_E_ were significantly reduced from 20–60 min after methadone in all groups **(B)**. At P0 and P1, methadone-induced frequency depression and *V*_T_ were blunted after MM compared to MN and MS **(B)**. Mixed-model ANOVA, Bonferroni *post hoc*, *MM *p* < 0.05 from MN, ^†^MM *p* < 0.05 from MS.

Next, we tested the hypothesis that MM desensitizes neonates to acute neonatal methadone-induced respiratory frequency depression. After daily neonatal methadone (1 mg/kg, i.p.), breathing frequency and *V*_E_ were reduced at 20–60 min in all maternal treatment groups at all time points after acute methadone (*p* < 0.05), with the exceptions of MM and MN P0 neonates at 20 min. For neonates after MS or MN, acute neonatal methadone-induced respiratory frequency depression was not different across ages. However, methadone-induced respiratory frequency depression after MM was blunted at P0 compared to P3–5 at 20 min and P4 at 40 min (*p* < 0.05). Methadone-induced respiratory frequency depression after MM was also blunted in P1 compared to P3–5 neonates at 20 min (*p* < 0.001), P2–5 at 40 min (*p* < 0.001), and P3–4 at 60 min (*p* < 0.05) (Figure 5). Between groups, methadone-induced respiratory frequency depression was blunted at P0 in MM compared to MS and MN, and at P1 compared to MS (Figures 5A,B). MM neonates also had a blunted depression in frequency in response to acute neonatal methadone at P2 compared to MN at 40 min (*p* = 0.021). By P3, methadone-induced respiratory frequency depression was similar for all groups (Figure 5B). A small, but insignificant, compensatory increase in *V*_T_ occurred in MS and MN neonates relative to MM neonates. However, tidal volume was increased at P1 in MS neonates relative to MM at 20 min (Figures 5A,B). Despite differences in methadone-induced frequency depression, methadone-induced depressions in *V*_E_ were overall similar for all maternal treatments likely due to compensatory increases in *V*_T_ (Figure 5B).

### Quantal Slowing Emerges at P2, Regardless of Maternal Treatment

**FIGURE 6:**
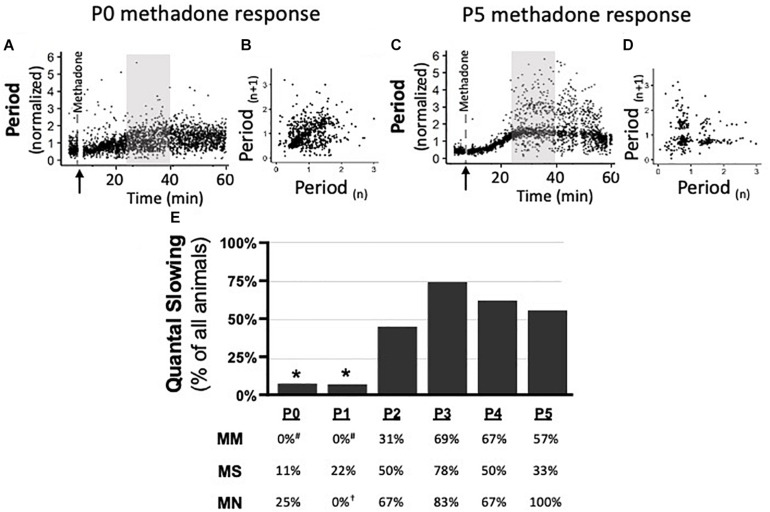
During neurorespiratory development, quantal slowing emerges at P2, regardless of maternal treatment. Representative changes in breathing period at P0 **(A,B)** and P5 **(C,D)** after acute neonatal methadone (1 mg/kg, i.p.; **↑**) demonstrate age-dependent methadone-induced frequency depression. Gray highlighted regions **(A,C)** after methadone injection shown as Poincaré plots (**B,D**, respectively) to visualize quantal slowing. Quantal slowing is evident as periods clustered at quantal integers of the normal respiratory period at P5, but not P0. Group data highlight that, regardless of maternal treatment, quantal slowing emerged at P2 in the majority of animals **(E)**. One-way ANOVA (graph), mixed-model ANOVA (table) Bonferroni *post hoc*, *p < 0.05 from P2 to P5, ^#^*p* < 0.05 from MM P3 to P5, ^†^*p* < 0.05 from MN P5.

Quantal slowing is the slowing of respiratory frequency by integer multiples of the normal breathing period and reflects mutual coupling between respiratory oscillators (Mellen et al., 2003; Janczewski and Feldman, 2006). Quantal slowing after acute methadone was observed in Poincaré plots during 15 min of peak respiratory frequency depression (25–40 min post-methadone injection, Figures 6A,C). Poincaré plots (Figures 6B,D) demonstrated either distributed slowing of respiratory period in younger neonates (P0 methadone response, Figures 6A,B) or quantal slowing in older neonates, evident as distinct groupings of breaths at integer multiples of the normalized period (P5 methadone response, Figures 6C,D). Regardless of maternal treatment, quantal slowing was significantly reduced at P0 and P1, but emerged at P2 (Figure 6E). The percentage of animals exhibiting quantal slowing from MM, MS, and MN groups highlights the age-dependency of the emergence of quantal slowing (Figure 6E). In MM neonates, quantal slowing was significantly reduced at P0 and P1 compared to P3–5 (*p* < 0.05). While in MN neonates, quantal slowing was significantly reduced at P1 compared to P5 (*p* < 0.001).

## Discussion

As the use of opioids during pregnancy increases (Epstein et al., 2013; Krans et al., 2015; Stover and Davis, 2015), improving our understanding of how the neurorespiratory system develops in the presence of opioids is a critical step in understanding how to best treat infants with NAS. Here, we show late gestation MM destabilizes early neonatal respiratory rhythm by increasing apneas and acutely desensitizes neonates to neonatal methadone-induced frequency depression. The respiratory rhythm instability after MM was most severe in the first day of life and normalized within the first 2 days. Contrary to our hypothesis, repeated daily acute neonatal methadone after MM did not significantly alter baseline breathing. However, MM desensitized neonates to acute neonatal methadone-induced respiratory frequency depression at P0–1. Interestingly, the desensitization normalized rapidly between P1 and P2, the same age quantal slowing emerged. These data suggest reorganization of respiratory rhythm generating circuits at P1–2, regardless of maternal treatment. Overall, MM destabilizes the neurorespiratory control system immediately after birth; however, the neurorespiratory control system is able to compensate and normalize breathing over postnatal development. Thus, an initial window of increased vulnerability to apneas exists immediately after MM, highlighting a window where treatment or increased monitoring is likely necessary.

A key aspect of the experimental design of this study is examining maternal opioids at a specific, critical time for vital respiratory neural activity onset to investigate impairment of maturation (circuit connectivity and refinement). Maternal opioids are known to impair CNS development, neuro- and gliogenesis, myelination and proliferation in other brain regions (reviewed in Hauser and Knapp, 2018); however, the majority of studies focused on maternal opioid exposure from the day of conception through birth. While previous studies provide insight into the effects of maternal opioids on cellular genesis, migration, apoptosis, and dysregulation of the neuroendocrine system (Conradt et al., 2018), opioids are known to have more severe effects late in gestation, when the neural system controlling breathing is becoming active. Opioid transmission across the placenta increases with gestational age (Nanovskaya et al., 2008) and are retained in the placenta, increasing fetal exposure time (Kopecky et al., 1999). Further, opioids have a prolonged half-life in the fetus (Scott et al., 1999), due to decreased pharmacokinetic clearance and excretion, decreased abundance of fatty tissues, and decreased receptor expression and sensitivity early in life (Dysart et al., 2007; Barr et al., 2011; Kocherlakota, 2014), suggesting maternal opioids may more severely impact *maturation* of neural circuits rather than cell genesis. Thus, this late gestation opioid exposure model sheds light on the direct effects of opioids on maturation of respiratory circuitry and allows us to disentangle the effects of opioids on cell genesis and migration from the maturation of critical network activity. Further, this model demonstrates for the first time that even late gestation opioids impair the respiratory control network. This model, however, was not designed to assess the amount of opioids transmitted through the breast milk, nor identify the specific age (between E17 and P5) where opioids have the most profound impact on respiratory network maturation. We hope future studies involving cross fostering of neonates between maternal treatment groups will begin to provide such insight.

Many different opioids are used clinically (e.g., methadone, morphine, buprenorphine) (McQueen and Murphy-Oikonen, 2016), but methadone [a long-lasting, complete μ-opioid receptor agonist (Kraft et al., 2016)] is the most frequently prescribed opioid to pregnant women (Kocherlakota, 2014; Davis et al., 2018). Methadone was given daily to pregnant rats from E17 through the first week after birth (Figure 1), whereby it will cross the placenta (Conradt et al., 2018) and continue to pass to the infant via the breast milk (Kocherlakota, 2014; Kraft et al., 2016; McQueen and Murphy-Oikonen, 2016). While the concentration of opioids in breast milk remains controversial (Kocherlakota, 2014), breastfeeding is encouraged for NAS infants (Kocherlakota, 2014). This model of a single daily injection best represents the initial “high/rush” of opioids, followed by the withdrawal experienced in addicts (Hauser and Knapp, 2018), and has been hypothesized to have the greatest impact on CNS maturation (Hauser and Knapp, 2018). As neonates are clinically treated with exogenous opioids after birth to reduce NAS symptoms (Kocherlakota, 2014; Kraft et al., 2016; McQueen and Murphy-Oikonen, 2016), neonates were also supplemented with methadone to investigate the additive effects of maternal and neonatal opioid exposure.

Maternal methadone increased the prevalence of apneas and post-sigh apneas, and destabilized breathing rhythm in neonates. Though the origin of apneas cannot be identified in this study, the prevalence of apneas suggests dysfunction of the respiratory control network maturation after MM. This dysfunction could be a result of concentrated neonatal methadone since opioids are known to be higher in fetal tissues compared to maternal levels (Peters et al., 1972) and opioids inhibit rhythm generating regions (Montandon et al., 2011). Neonatal CNS methadone levels can even exceed maternal levels as methadone accumulates in fetuses (Peters et al., 1972; Peters, 1975), likely due to blunted opioid metabolism in neonates. Furthermore, maternal opioids continue to be transmitted to neonates through breast milk (Hendrickson and McKeown, 2012) and some evidence suggests human neonatal apneas are the result of opioid exposure through maternal milk (Naumburg and Meny, 1988; Anderson et al., 2016a; Cleveland, 2016). Therefore, MM may increase apnea prevalence as a result of high concentrations of methadone impairing development of neonatal respiratory neural circuitry. However, while concentrated methadone could induce apneas in neonates, methadone suppresses sighs (Bell et al., 2011), which were elevated after MM in P0 neonates. Therefore, elevated neonatal methadone levels alone are unlikely to explain both the increase in apneas and increase in sighs; and thus, we suggest likely reflects impaired development of respiratory neural circuitry. Since apneas are more common in preterm or very early neonatal infants (Greer, 2012), MM could alternatively delay neurogenesis (Wu et al., 2014) and alter myelination (Sanchez et al., 2008; Walhovd et al., 2010; Vestal-Laborde et al., 2014; Hauser and Knapp, 2018). Therefore, MM may delay respiratory rhythm generating network maturation as maternal treatments began at E17, the day respiratory rhythm begins (Greer, 2012). A relatively immature respiratory control system could also explain the increased prevalence of apneas after MM. Lastly, the effects of opioids on other aspects of the respiratory control network could also contribute to these deficits in breathing, including regions influencing pattern and rate (e.g., Kölliker-Fuse, locus coeruleus, and post-inspiratory complex) (Anderson et al., 2016b; Kliewer et al., 2019; Saunders and Levitt, 2020; Varga et al., 2020a,b). Future studies should focus on identifying the mechanisms of these breathing deficits and involvement of different aspects of the respiratory control circuitry.

Maternal methadone alone did not significantly reduce neonatal weight gain. Other reports (McLaughlin and Zagon, 1980; Byrnes and Vassoler, 2018) suggest longer MM treatments reduce neonatal weights, suggesting our exposure starting on E17, during a critical period for neurorespiratory development, is not inducing global developmental deficits. However, in combination with daily acute neonatal methadone, neonatal weights after MM were reduced. Similarly, human infants withdrawing from methadone gain less weight in the first week of life despite hyperphagia, suggesting altered metabolism after acute methadone (Martinez et al., 1999). Since we did not assess metabolism in our studies, it is unclear if MM alters neonatal rat metabolism. Further, it is unlikely MM induces long-lasting deficits in weight gain since adult weights are normalized in other studies with longer MM treatments (McLaughlin and Zagon, 1980). However, it remains unclear if adult weights are impacted by the combination of MM and daily acute neonatal methadone.

Peak hypoxic responses were unaffected by MM, suggesting the respiratory control network can still respond appropriately to hypoxic stimuli. The phase I, peak hypoxic ventilatory response is typically attributed to activation of the peripheral chemosensors in the carotid body (Fung et al., 1996), which express opioid receptors (Ricker et al., 2015) and are inhibited by opioids in adults (Ricker et al., 2015). However, acute opioids also depress hypoxic responses through direct actions on the brainstem (Bailey et al., 2000; Pattinson, 2008), suggesting balancing of peripheral and central effects. Thus, the results presented here suggest MM does not alter carotid body function. Regardless of maternal treatment, the phase I ventilatory response to hypoxia increased substantially in the first 6 days of life, similar to previous reports of HVR development (Liu et al., 2006).

Unlike phase I, the phase II hypoxic ventilatory decline was significantly attenuated after MM. Phase II is known to be most severe in young neonates (Bissonnette, 2000; Moss, 2000; Liu et al., 2006) and is mediated by more complex mechanisms, likely involving changes in metabolism during hypoxia and brainstem signaling (Bissonnette, 2000). Purinergic signaling is significantly involved in hypoxic ventilatory depression, especially in young neonates (Elnazir et al., 1996; Gourine et al., 2005; Rajani et al., 2018), though it remains controversial (Funk and Gourine, 2018). The role of purinergic signaling in perinatal rats changes substantially from fetal to neonatal ages in rats (Huxtable et al., 2009), suggesting MM may alter the normal development of the purinergic system to blunt the hypoxic ventilatory decline after MM at P0 and P1. Further, the phase II response after maternal opioids is similar to the response in more mature animals; thus it is possible that opioids also modulate the developmental trajectory of these networks, and a blunted phase II response may be the signature of accelerated development. Interestingly, and contrary to our findings, human infants from mothers suffering from polysubstance abuse had significantly greater acute hypoxic responses and greater hypoxic ventilatory decline in the first week of life (Ali et al., 2016). However, these effects are confounded by the intake of multiple different types of drugs, which may independently alter development (Nettleton et al., 2008). Thus, MM blunts the hypoxic ventilatory decline in neonatal rats, though whether this is physiologically advantageous or disadvantageous remains to be determined.

Maternal methadone did not alter HCVR, suggesting the central networks sensing CO_2_/pH are not altered by MM. Unlike this response in neonatal rats, MM or morphine enhances the HCVR in guinea pigs (Nettleton et al., 2008). Contrary to our and other animal findings, human infants chronically exposed to prenatal methadone have significantly blunted HCVRs, which persist for the first two weeks of life (Olsen and Lees, 1980). These differences in the HCVRs may be explained by species differences, the duration, or timing of MM exposure. Our paradigm had only 5 days of MM, while the others last for at least half of gestation, suggesting earlier developmental exposure to methadone may have differential effects on hypercapnic responses.

Maternal methadone had no significant effect on baseline breathing responses to daily, repetitive acute neonatal methadone, but desensitized P0–1 neonates to acute neonatal methadone-induced respiratory frequency depression. There are at least three explanations for these changes in opioid sensitivity. Firstly, MM could downregulate expression of opioid receptors in neonates, thereby reducing preBötC sensitivity to opioids from P0 to P1 and blunting methadone-induced respiratory frequency depression. Maternal buprenorphine downregulates μ-opioid receptors in P1 rat whole brains (Belcheva et al., 1998), demonstrating expression of μ-opioid receptors is influenced by gestational opioids. Additionally, μ-opioid receptors normally increase in the rat brainstem from P3 to P6 (Kivell et al., 2004) and, as opioid receptor expression increases, methadone-induced respiratory frequency depression likely also increases, potentially explaining the increase in methadone-responses at P3 after MM. Secondly, competing inhibitory and excitatory actions of opioids in different respiratory regions could explain the blunted respiratory frequency depression at P0–1 after MM. While the preBötC rhythm is inhibited by opioids, the pFRG rhythm may be facilitated by opioids in P0–2 rats (Tanabe et al., 2005; Onimaru et al., 2006). Therefore, increased pFRG excitation could counteract depression in the preBötC to blunt methadone-induced frequency depression. Third, early exposure to methadone could impair preBötC activity during development, such that the respiratory network is more reliant on pFRG rhythm generation in the first 2 days of neonatal life. Thus, a rhythm generating network dominated by the pFRG would be less sensitive to opioid-induced respiratory frequency depression. Future experiments assessing methadone responses of isolated respiratory circuitry and the developmental expression of opioid receptors will delineate how MM alters methadone-induced respiratory frequency depression.

Interestingly, only MM treated neonates exhibited differential methadone responses over time, due to their blunted acute neonatal methadone sensitivity at P0–1. Previous studies are conflicting on the development of opioid-induced frequency depression in rodents. One study demonstrates early μ-opioid receptor-induced respiratory frequency depression is blunted at P1 relative to P10 (Greer et al., 1995), while another suggests respiratory frequency depression is similar between P2 and P8 (Colman and Miller, 2001). Here, methadone sensitivity was unchanged over time in neonates from maternal saline and maternal no treatment groups, suggesting no developmental shifts in opioid sensitivity. However, we used repetitive daily methadone injections in the same rats from P0 to P5 and, in many other physiological systems, opioids induce tolerance within the first few exposures in rodents (Ling et al., 1989). Tolerance in the respiratory system has been observed after daily acute fentanyl (Laferrière et al., 2005), though this has not been observed in other studies (Emery et al., 2016; Levitt and Williams, 2018). Here, we found no evidence for tolerance after repeated daily acute methadone exposure, but we demonstrate the unique finding that opioid sensitivity is blunted acutely after MM. Furthermore, it is widely thought the respiratory control system does not develop opioid-induced tolerance (Emery et al., 2016; Levitt and Williams, 2018), which increases vulnerability of patients to lethal respiratory frequency depression with opioid over-use. Thus, the demonstration of blunted opioid sensitivity represents a unique finding regarding sensitivity of developing neurorespiratory networks to opioids.

The neonatal period (P0–5) is an important developmental time for respiratory rhythm generating mechanisms during which the dominance of the two respiratory oscillators switches (Huckstepp et al., 2016). However, the precise timing of the switch is not clear. The phenomenon of quantal slowing represents a physiological means to probe the functional interactions of these two oscillators, whereby quantal slowing occurs when the two oscillators are in phase (Wittmeier et al., 2008) and the preBötC is the dominant inspiratory rhythm generator. Regardless of maternal treatment, quantal slowing emerged in the majority of rats at P2. This change likely reflects a network reorganization at this age irrespective of maternal treatment and suggests a strengthening in connectivity between the pFRG and preBötC. Previous work demonstrates pFRG activity precedes and drives preBötC activity in P0–1 rats (Onimaru and Homma, 2003) and then synchronizes with preBötC activity at P2 (Oku et al., 2007), suggesting quantal slowing may only emerge when the two oscillators’ activities are tightly coupled. Additionally, the changes in this coupling of activity may be related to the considerable maturation of neurotransmitter systems occurring at this developmental stage. These changes include large changes in chloride conductance and its inhibition of respiratory rhythm (Ren and Greer, 2008), increased preBötC GABA receptor expression at P2 (Liu and Wong-Riley, 2004), and changes in AMPA receptor subunit expression (Wong-Riley and Liu, 2008). Such changes may strengthen connections between the preBötC and pFRG, coupling their activity, and contributing to the emergence of quantal slowing at P2. The emergence of quantal slowing and shifts in functional connectivity between oscillators may have important implications for interpreting rhythm generating mechanisms at different neonatal ages. Specifically, the majority of studies on respiratory rhythm generation occur in neonatal rodents in the first week of life; however, the precise age range varies between and within studies. Therefore, investigations of respiratory circuitry before and after P2 may reveal divergent results and should be interpreted with these developmental changes in mind.

In our studies, MM treatment began on E17 because it is the first day of rhythmic respiratory neural activity, respiratory motor output, and fetal breathing movements in rats (Greer, 2012). While other studies focused on the developmental impact of maternal opioids begin earlier to match the period when opioid receptors are first expressed in rodents (Byrnes and Vassoler, 2018), our experimental paradigm was intentional to mitigate any other developmental effects of methadone and focus on this important period of maturation of neurorespiratory development. Methadone was chosen since it is commonly used as a clinical treatment in pregnant women and neonates (Kraft et al., 2016; Pryor et al., 2017; Bhavsar et al., 2018; Davis et al., 2018). However, methadone is also a non-competitive NMDA antagonist (Laurel Gorman et al., 1997), which may contribute to opioid-induced respiratory frequency depression (Hoffmann et al., 2003). Furthermore, mice lacking NMDA receptors have more apneas in the first few days of life (Poon et al., 2000). However, central respiratory rhythm-generating networks develop normally in the absence of NMDA receptors (Funk et al., 1997) and the increase in apneas may be due to *in utero* alterations to mechanosensory, chemosensory, or pontine respiratory areas (Funk et al., 1997; Poon et al., 2000). Since we did not determine the source of apneas in this study, it is unclear if methadone antagonism of NMDA receptors is contributing to early-life respiratory instability.

Endogenous opioid systems modulate neonatal breathing (Jansen and Chernick, 1983) and may be altered by chronic methadone exposure. A surge of endorphins shortly after birth in neonatal brains (Jansen and Chernick, 1983) inhibits the preBötC, causing the pFRG to acutely dominate respiratory rhythm and maintain breathing rhythm (Feldman and Del Negro, 2006; Janczewski and Feldman, 2006). It is unclear if MM changes the endogenous opioid system to impair respiratory instability at P0 or blunt early insensitivity to acute methadone. However, it is unlikely alterations in the endogenous opioid system explain the results presented here since MM in rats did not alter the endogenous opioid system in the neonatal rat ventral respiratory column (Gourévitch et al., 2017).

Understanding how maternal opioids alter neurorespiratory development is important for enhancing treatments for neonates exposed to *in utero* opioids. Our data support the hypothesis that MM increases apneas and destabilizes neonatal breathing. This instability may be clinically relevant as apneas are more common in infants dying of sudden infant death syndrome (SIDS) (Kahn et al., 1992; Franco et al., 2003) and MM increases the risk of SIDS (Moon, 2016). Additionally, the hypoxic and HCVR were unaffected by maternal treatment. In humans, however, MM depresses hypoxic (Ward et al., 1992) and hypercapnic responses (Olsen and Lees, 1980), suggesting differences in species or duration of *in utero* methadone exposure are important. MM also blunted the acute response to neonatal methadone, suggesting neonates with NAS may have less respiratory frequency depression after acute opioid treatments. Therefore, NAS infants potentially tolerate higher acute therapeutic doses to mitigate other symptoms of NAS, though the long-term impact of perinatal opioids remains to be determined. This protection from opioid-induced respiratory frequency depression after acute methadone is lost rapidly in neonatal rats at P2, likely at the same time as the preBötC becomes the dominant respiratory rhythm generator. It is not clear if humans have a similar developmental trajectory of multiple respiratory rhythm generators as rodents. Importantly, the results presented here demonstrate neurorespiratory disruption by MM, which may be important for understanding and treating the increasing population of neonates exposed to gestational opioids.

## Data Availability Statement

The raw data supporting the conclusions of this article will be made available by the authors, without undue reservation.

## Ethics Statement

The animal study was reviewed and approved by the Institutional Animal Care and Use Committee (#18-02), University of Oregon. This study was performed in strict accordance with the recommendations in the Guide for the Care and Use of Laboratory Animals of the National Institutes of Health.

## Author Contributions

AH, NM, and AHu contributed to interpretation, drafting and revising critically for important intellectual content. MS also contributed to drafting and revising critically for important intellectual content. AH, NM, and MS contributed to acquisition and analysis of data. AHu contributed to conception and design of the work. All authors contributed to the article and approved the submitted version.

## Conflict of Interest

The authors declare that the research was conducted in the absence of any commercial or financial relationships that could be construed as a potential conflict of interest.
